# Morpho-Structural Investigations and Carbon Nanoclustering Effects in Cr-Al-C Intermetallic Alloys

**DOI:** 10.3390/nano12183225

**Published:** 2022-09-16

**Authors:** Alina Daniela Crisan, Ovidiu Crisan

**Affiliations:** National Institute for Materials Physics, P.O. Box MG-7, 077125 Magurele, Romania

**Keywords:** ternary alloys, crystallization, annealing, X-ray diffraction, carbon nanoclustering

## Abstract

Intermetallic Cr-Al-C thin films from the 211 class of MAX phases were fabricated via ion beam deposition and structural investigations were undertaken to obtain information about morpho-structural effects propelled by carbon excess in the stoichiometry of the films. In order to promote the occurrence of the Cr_2_AlC MAX phase, the stoichiometric thin films were subsequently annealed at two temperature values: 650 °C and 700 °C in UHV conditions for 30 min. The morpho-structural effects in both as-deposited and annealed films were monitored using scanning electron microscopy, X-ray diffraction, and Raman spectroscopy. XRD analysis showed that the as-deposited sample was almost completely crystallized in the hexagonal Cr_2_AlC structure, with a remaining amorphous fraction of about 17%, most probably rich in carbon. Raman analysis allowed the identification of three spectral regions, two of them encompassing the Raman optical modes belonging to the Cr_2_AlC 211 MAX phase, while the third one gave strong evidence of highly intense and large D- and G-bands of carbon. Structural parameters such as the crystal lattice parameters as well as the volume of the crystal unit cell were found to decrease upon annealing; this decrease is attributed to the grain growth. The average crystallite dimension was proven to increase after annealing, while the lattice micro-strain lowered to approximately 63% in the annealed thin film compared to the as-deposited one. Well-formed and intense Raman peaks attributed to D- and G-bands of carbon were also observed and, corroborated with the structural data, seemed to indicate an overall increased level of crystal ordering as well as potential carbon nanoclustering after thermal treatments with thin Cr_2_AlC films. This observed phenomenon concords with previously documented reports on ab initio modelling of possible Cr_2_AlC structures with carbon excess.

## 1. Introduction

Intermetallic ternary compounds Cr-Al-C belong to generic MAX systems, albeit carbides and nitrides, where M is a transition metal, A is a metalloid, and X is either C or N. Initially documented in the 1960s by Jeitschko and Novotny [1] and revamped by Michael Barsoum et al. [2], these systems have attracted very large interest lately, as they have proven to exhibit both metallic and ceramic features. The MAX phase class represents the parent system from which the hugely popular MXene category has lately arisen, a category that comprises transition metal binary carbides and nitrides synthesized in the form of nanosheets in a procedure similar to ones for graphene synthesis. They exhibit high electric and thermal conductivity, they are easily machinable, very hard, and have good resistance to oxidizing phenomena and sudden changes in temperatures. Synthesised as thin films, these alloys have potential applications as hard coatings for magnetic bearings, magnetic levitation, superconducting magnetic energy storage systems, and other machine parts which work in high T environments.

The structure of the Cr-Al-C is what determines its very promising physical properties. As shown in several papers [3,4,5,6,7], Cr_2_AlC crystallizes in a hexagonal structure, having the space group *P63/mmc*, with a hexagonal crystal unit cell made of two Cr_6_C octahedra intertwined with a slab of Al atoms. The alloy material Cr_2_AlC was reportedly fabricated as thin films using magnetron sputtering from individual elemental targets [4,5] as well as from targets of Cr-Al compounds with C addition [6,7,8,9]. This particular high vacuum alloying method was successfully used for synthesizing other nanocrystalline compounds and intermetallic alloys [10,11,12]. For instance, by sputtering from a compound target, Walter et al. [6] obtained thin films of single-phase Cr_2_AlC deposited on steel which was previously heated at 650 °C. The investigation of this MAX phase system continued over the years, and currently, there is a tremendous research effort among materials scientists to investigate the Cr_2_AlC system for its potential applicability in a variety of technological fields. Cr_2_AlC was studied as a novel saturable absorber to produce a nanosecond pulse Q-switched fiber laser [13] or for its effectiveness as electromagnetic interference shielding materials [14]. Their bulk sintered form was also investigated for several industrial applications [15]. The influence of the synthesis conditions on the structure, tribology properties, corrosion, and oxidation resistance was also investigated [16,17,18]. Some studies also dealt with the effects of annealing Cr_2_AlC films by ion irradiation [19].

In our previous study [20], we successfully obtained a single 211 MAX phase in thin films from the Cr-Al-C ternary system at even lower than previously reported temperatures: approximately 450 °C. In another work, we also proved that the elastic and structural behavior of hard coatings made of Cr-Al-C [21] are highly influenced by re-crystallization procedures during in-air annealing, which promotes supplemental corrosion protection by forming a Cr oxide protective layer on the surface of the films.

In the present work, we investigate the morpho-structural effects in the Cr-Al-C films obtained via deposition by sputtering onto Si(100) at 200 °C, and we also study the structural effects induced by subsequent thermal treatments on the resulting films. This study is driven by the possibility of using such nanofilms as coatings of magnetic bearings and also in superconducting magnetic energy storage applications due to their possible carbon nanoclustering effects, where excess carbon may create low friction intergranular layers.

The advantages of nanocoating the magnetic bearings to enhance the surface properties and overall operation performances of magnetic bearings have been pointed out by T.Y. Cho et al. [22], who demonstrated that hard coatings made of carbides such as WC, followed by laser annealing, contribute strongly to the increased surface hardness of the magnetic bearing. The main reason for this improvement was linked to the partial decomposition of carbon excess through laser annealing, thus forming a rather porous interface functioning as a buffer zone and increasing the overall adhesion of the coating. As a different approach, F. Sass et al. [23] proved the efficiency of coating superconducting wires for the bearings used in the magnetic levitation vehicles of the future, as well as for superconducting magnetic energy storage.

In several works regarding thin films of Cr-Al-C and generally on MAX phases, for instance, in ref. [24], it was shown that there are some disadvantages when using coatings made of MAX-phase alloys, one of these being linked to the high temperature required to heat the substrates in order to form the MAX phase, regularly above 650 °C, values that usually prohibit industrial applications and restrain the choices for substrates to only materials stable beyond 650 °C. In this view, the procedure of annealing applied subsequently after the deposition procedure may ensure better crystallization of the as-deposited films, even in ambient conditions, with the oxidation processes severely limited.

The present work investigates the overall structural properties of Cr-Al-C films via a complex morpho-structural characterization of both as-deposited and annealed samples.

## 2. Materials and Methods

Thin films of Cr-Al-C were synthesized by ion-beam sputtering in a custom-made ultra-high vacuum chamber with an initial pressure of 2 × 10^−6^ mbar. The custom-made chamber has two ion beam sources, one which is vertically mounted, which is used for sputtering the target and a second one which is horizontally mounted. The second ion source acts for ion assisting the deposition, a procedure which ensures uniform ion beam flux, allowing a smoother process of deposition of the film on the substrate. The composite target was made of a pure Cr (99.99%) plate, which was partially covered by graphite and Al sheets. Areas of the different elemental targets are fully adjustable to allow fine changes in the stoichiometry of the deposited films. The composite target was sputtered using an ion beam of Ar+ ions of 1.2 keV energy using a current of 125 mA and an acceleration voltage of 1.5 kV, a beam furnished by a top-mounted 75 mm Kaufman ion source. The thin films were sputtered onto Si (100) substrates inside the chamber with an Ar atmosphere of 2.25 × 10^−4^ mbar. Prior to deposition, the target Ar+ ion was sputter-cleaned for 1 h. The stoichiometry of the deposited films was checked using Energy-Dispersive X-ray Spectroscopy (EDX). The crystal structure and the surface morphology of the as-deposited and laser-treated films were studied using scanning electron microscopy (SEM) and Raman spectroscopy. Scanning electron microscopy images were recorded with a confocal device microscope (EVO 50 XVP purchased from Carl Zeiss GmbH, Oberkochen, Germany). XRD studies were performed using a powder diffractometer (D8 Advance from Bruker AXS GmbH, Karlsruhe, Germany) with Kα radiation of Cu (λ = 0.154 nm). The X-ray data were obtained in a geometry θ–2θ in an angular interval of 20° to 90°. Important structural parameters were devised from a full-profile analysis of the obtained XRD spectra using MAUD software (Materials Analysis Using Diffraction, version 2.99, University of Trento, Italy). Raman spectroscopy was performed with a Renishaw InVia (Renishaw plc, Gloucestershire, UK) spectrometer, with data collection in a backscattering configuration. For the Raman backscattering experiments, an Argon laser (λ = 512 nm) was employed to irradiate the sample surface using a 50× objective, producing a spot of approximately 830 nm in diameter. The signal was collected using an air-cooled CCD camera. The spectral resolution of the obtained spectra was below 2 cm^−1^. During the experiments, the laser power was adjusted in order to provide tuneable analysis conditions. The spectra were subsequently fitted using a multi-peak pseudo-Voigt fitting function for the whole profile. For this purpose, we employed a peak fitting procedure with the help of dedicated software, especially developed for spectral peaks deconvolution (PeakFit 4.12 Software, from Systat Software GmbH, Erkrath, Germany).

## 3. Results and Discussion

### 3.1. Composition and Morphology

For the synthesis of the thin films, the dual ion beam procedure of deposition in a UHV chamber was employed to obtain the as-deposited samples with a constant thickness of approximately 150 nm. Adjustable synthesis parameters allow us to obtain different compositions for ternary compound thin films. The resulting compositions were checked using the EDX module during the scanning electron microscopy observations of the samples. The composition of the sample, resulting from EDX measurements, was found to be Cr_47_Al_27_C_26_ in weight percentages. The estimated stoichiometry errors, averaged over all three determinations, were approximately 0.15 wt%. Several annealing treatments were performed on the as-deposited sample. These annealing treatments were performed in the UHV chamber at various temperatures: 650 °C and 700 °C, respectively, in ultra-high vacuum conditions (10^−6^ torr).

**Figure 1:**
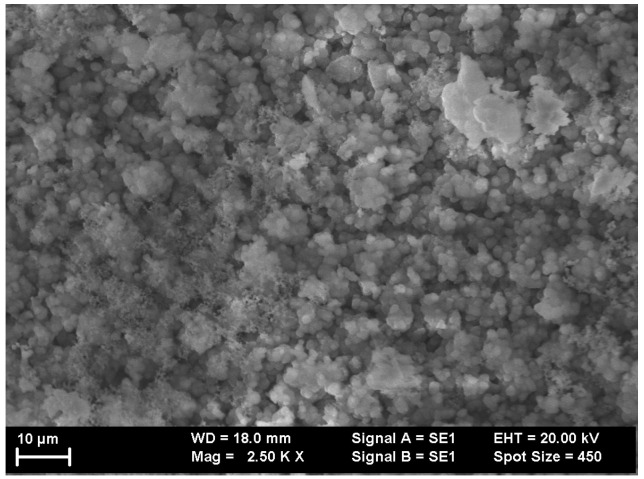
The surface of Cr_47_Al_27_C_26_ as-deposited thin film, as revealed by the scanning electron microscopy images.

**Figure 2:**
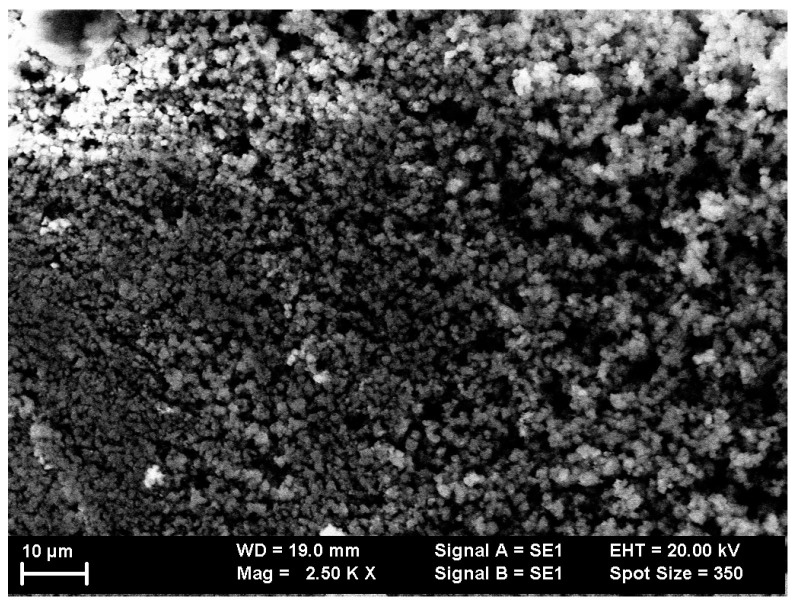
The surface of Cr_47_Al_27_C_26_ thin film annealed at 700 °C, as revealed by the scanning electron microscopy images.

For morphology investigations of the thin films, SEM images were recorded on the surface of both as-deposited and annealed samples. Figure 1 and Figure 2 present images of the sample Cr_47_Al_27_C_26_ as-deposited and annealed at 700 °C.

On analyzing the SEM images, it can be inferred that the morphology is quite irregular at the surface, with cluster-like growth accompanied by the aggregation of multiple grains. The as-deposited sample presents some large clusters of grains with a mean diameter of roughly 10–12 μm. A slightly different image was provided by the annealed sample. In this case, a much smaller average grain size was observed, at approximately 5–8 μm. The annealed sample presented evidence of vertical clustering in dendrites, whereas in the as-deposited film, its surface presented columnar-like aggregation. The contrast of the recorded images was similar to the features observed, with no other different texturing observed. This proves the homogeneous nature of the deposition accompanied by the lack of unalloyed metal species at the surface and secondly, that most probably all features correspond to a single crystalline phase Cr_2_AlC or 211 MAX phase. By averaging the observed grains, it was possible to calculate the grain size distributions. These were found to be centered around 11 ± 0.45 μm and 6 ± 0.27 μm for the as-deposited sample and annealed sample, respectively.

### 3.2. Raman Analysis

#### 3.2.1. Peak Assignation

Raman spectroscopy was used to analyze the surface of the Cr-Al-C thin films. For that purpose, unpolarized Raman scattering spectra were collected at room temperature from the surface of the films. The investigated range of the wavenumber in the analysis was within 120 ÷ 2000 cm^−1^, and in our experiment, three typical regions were identified, characteristic of the structural effects in MAX phases. It must be noted that the MAX phase structure typically belongs to the D^4^_6h_ space group. For the lower symmetry component of the MAX phases, the 211 structures, it was reported [25,26] that the system comports four optical modes, of which three are solely Raman-active, and the remaining one is both Raman- and infrared-active [27]. These optical modes were scattered over all of the investigated intervals.

We previously inferred [21] that there are three important regions for low, intermediate, and high wavenumbers in the spectra. However, in the existing literature, only one or two of these Raman regions are usually covered. While the first region is the most investigated in the literature, the full spectrum analysis for MAX phases over all three regions is novel in our study. For instance, Spanier et al. [25] explored the 312 MAX phases until only 800 cm^−1^, while Lin et al. [28] presented Raman peaks in Cr_2_AlC only between 1200 and 1500 cm^−1^, which are not attributable to the MAX phases themselves but to the nanoclustering carbon bands, as thoroughly explained hereafter.

The first spectral region, for wave numbers between 120 and 400 cm^−1^, comprises general emission lines that are typical for M_2_AX phases and may constitute the distinctive signature of the presence of that particular phase in the structure of the film. As mentioned before, the 211 structures belong to the space group D^4^_6 h_. The three optical modes that are solely Raman-active correspond to *A_g_* + 2*E*_2*g*_ levels, and there is a fourth one which is both Raman- and infrared-active (*E_g_*) [25]. The peaks identified in the first spectral region are of high intensity and quite sharp, are denoted as *1a–1d* peaks, and are usually attributed to the optical modes assigned to MAX phases. The Raman peak attribution is consistent with previously reported studies on Cr_2_AlC thin films [25,26,27,28].

The second, intermediate, spectral region, between 550 and 900 cm^−1^, usually contains two broad peaks of lower intensity, denoted as *2a-2b*. These peaks are also observed in single-phase MAX films; therefore, their presence cannot be attributed to other binary phases formed in the film structure. These peaks may possibly be attributed to other Raman-active modes in the Cr_2_AlC phase. In this intermediate region, a very intense, sharp peak may sometimes manifest, especially in oxidized samples. This strong peak is customarily assigned to chromium oxides, notably Cr_2_O_3_, as suggested in ref. [20]; however, this was not manifested in our samples since the laser annealing procedures undertaken on our samples provided an oxygen-free environment, and therefore, the oxidation usually appearing in classic annealing was avoided overall.

The third spectral region, situated in the range 900 to 1800 cm^−1^, presents quite large and very intensive Raman lines, situated around 1345 and 1580 cm^−1^, respectively. In accordance with several other investigations [29,30], the large peaks are usually assigned to D- and G-bands of carbon, manifesting in the 211 films with carbon excess. Since thin films of disordered carbon are proven to consist of covalent mixtures of sp^2^- and sp^3^-bonded carbon, in the model proposed by Ferrari et al. [29], it was assumed that the line attributed to the G-band was due to the shortening of carbon bond in the pairs of sp^2^ atoms whereas the D-band peak might be due to the *A*_1*g*_ breathing modes. These modes are reportedly situated [29] at 1360 and 1560 cm^−1^, respectively. From investigations of Raman spectra recorded on diamond-like carbon thin films, using thermionic vacuum arc deposition, Musa et al. [30] also found very broad Raman peaks in this third spectral region, positioned to approximately 1380 cm^−1^ and 1560 cm^−1^, respectively. These peaks are attributed to the D-band and G-band of carbon bonding. Taking all these into account, we can safely conclude that in the case of our samples, the occurrence of the peak attributed to the G-band shows the nanoclustering of carbon after the annealing of the Cr_2_AlC thin films. However, as described in the literature [7,18,31], there are other potential explanations for the dominance of D- and G-bands attributed to sp2-bonded carbon, as reported in ref. [32], where it was suggested that large-size carbon clusters have preferential orientation. Additionally, it was argued in ref. [18] that these carbon-related spectral areas can be attributed to clusters of diamond-like carbon with wide boundaries of sp2-bonded carbon.

#### 3.2.2. Fitting Procedure

Due to all the aforementioned reasons, the Raman spectra of MAX phase materials are usually very complicated and are made of several complex-shaped peaks that sometimes overlap. Therefore, proper data analysis and peak separation are essential in order to analyze structural changes in MAX phase materials during processing and phase transformation. For our fitting, a pseudo-Voigt fitting function was chosen. The software allows for automatic Fourier domain filtration. Before the deconvolution procedure, the spectral background was removed from the experimental data.

**Figure 3:**
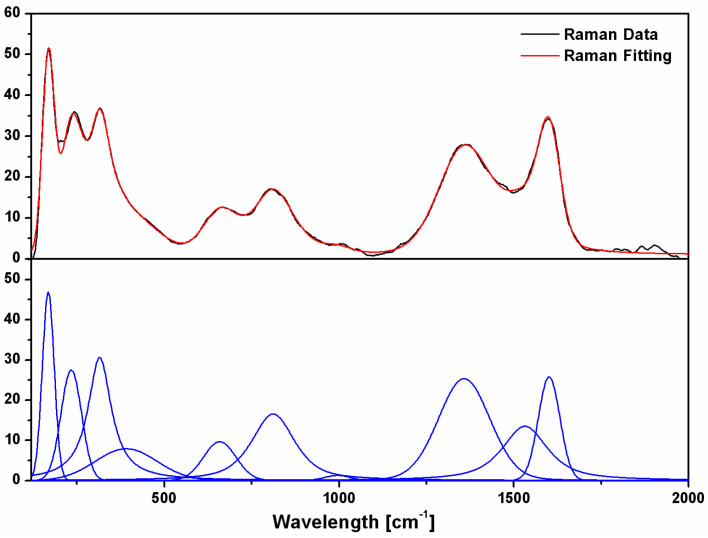
Example of fitting Raman spectra: top graph shows measured Raman spectra (black line) together with the best fit (red line); bottom graph shows the individual deconvoluted peaks resulting from the fitting.

To illustrate the procedure of fitting and its results on assessing the individual peaks of the experimental data, an example of a fitted Raman spectrum is presented in Figure 3. In the top graph, the measured Raman spectrum (black line) is compared with the best fit line (red line). It is obvious that the fitting line follows the experimental data quite closely, and it is thus proven that the fitting algorithm we chose provides quite accurate fitting of the experimental data. The bottom graph shows the individual peaks resulting from the fitting, components that were successfully deconvoluted from the overlapped peaks from the various regions of the Raman spectrum during the fitting procedure. In order to achieve these results, a Gaussian–Lorentzian area model was used that enables the deconvolution of peaks with variable widths, properly adjusting the widths and taking into account the peak asymmetry.

#### 3.2.3. Effects of Annealing

**Figure 4:**
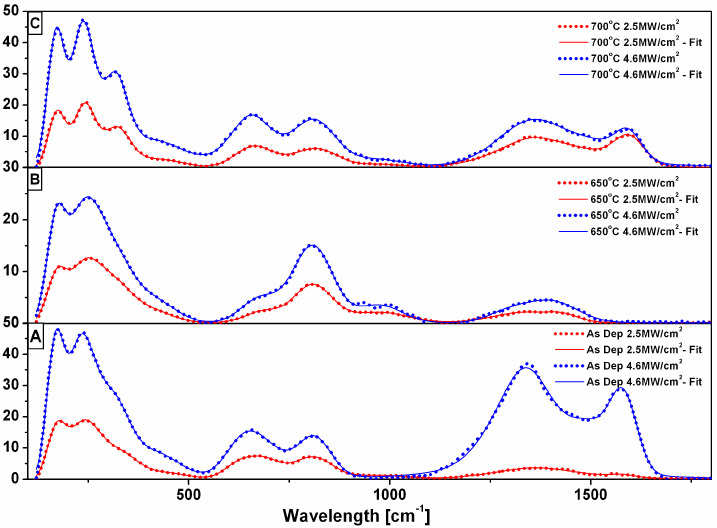
Raman spectra for sample no.1 at two different laser power values: 2.5 and 4.6 MW/cm^2^, respectively. (**A**) As-deposited, (**B**) annealed at 650 °C for 30 min, and (**C**) annealed at 700 °C for 30 min. Experimental data are represented by dots, while the data fitting is represented by the continuous line.

In order to assess the effects of annealing on the as-deposited films, several annealing treatments were performed on the as-deposited samples. Annealing was performed in the dedicated UHV chamber, where there are ideal, oxygen-free annealing conditions. The samples were annealed at various temperatures: 650 °C and 700 °C, respectively, in ultra-high vacuum conditions (10^−6^ torr). Raman results for this sample are shown in Figure 4. The graph labelled as A shows the Raman experimental data for as-deposited sample 1. The data were obtained for wavenumbers between 150 and 2000 cm^−1^ in two laser irradiation modes: high laser power density (h-LPD) and low laser power density (l-LPD). In the graphs, the three spectral regions detailed above are clearly delimited by intervals with no Raman peaks. All three regions contain delimited Raman peaks, according to the definition given above; however, the peaks are better formed in the case of the h-LPD irradiation mode. For the as-deposited films, the clear effect of the increased laser power in all three regions was observed (0–500 cm^−1^, 500–1000 cm^−1^, and 1000–1600 cm^−1^). Concretely, while the l-LPD mode excites the Raman optic modes only weakly, providing large, convoluted peaks with low intensities (very low in the case of region 3), the h-LPD mode excites the Raman optic modes quite strongly, providing Raman peaks of high intensities that are very well formed. The effect of increasing the laser power density was very prominent, especially in regions 1 and 3. Raman peaks *1a*–*1d* from region 1 were determined and deconvoluted via our fitting procedure with PeakFit. The Raman peaks from region 2 (*2a* and *2b*) were equally observed, with similar shapes, also in the l-LPD mode but with a lower intensity. In the case of region 3, on increasing the laser power density, there was a spectacular increase in intensity (ten-fold) for the two Raman peaks related to the carbon D- and G-bands. Therefore, it is obvious that the h-LPD mode produces significantly heightened evidence for excess carbon bonding, which may be related to the carbon segregation and nanoclustering in the material.

Upon annealing at 650 °C (Figure 4B) and 700 °C (Figure 4C), the Raman spectra show similar trends in the first region, between 0 and 500 cm^−1^, where the Raman peaks associated with the MAX phase were observed, as was the case in the as-deposited sample, with almost the same features in terms of peak width, shape, and intensity. However, the situation was radically changed, especially in the case of the third region. Here, it was noted that for 650 °C annealing, the D- and G-band related peaks, peaks that were quite significant in the as-deposited sample, almost disappeared. This disappearance could be related to the re-alloying of the excess carbon within the main MAX phase, associated with an increase in the crystal sizes due to the high energy furnished to the film surface during annealing. Upon further annealing at 700 °C, these Raman peaks from the third region, associated with carbon D- and G-bands, started to reform, most probably due to a process of carbon segregation within the MAX phase grain boundaries. This led to the establishment of an intergranular region which was carbon-rich and evidenced by the presence of the *3a*, *3b,* and *3c* Raman peaks, according to the above-mentioned definition. The Raman peaks associated with the MAX phase in the first regions were the best-formed and more clearly visible for the film annealed at 700 °C, where the structural refinement of the film led to better crystallization and larger crystal sizes.

All the values of the Raman peaks, together with their assignation, resulting from fitting, are listed in Table 1. It is shown that while for the first spectral region, the peak assignations concord with the results from the literature, for the second and third spectral regions, our analysis goes beyond the usually investigated range in the literature; therefore, available comparison data for these regions are scarce. For instance, in ref. [7], Raman measurements of CrAlC and CrAlCN were presented but with quite high noise and very low-intensity lines in intervals covering our defined third spectral region, and it was interpreted that the very broad Raman peaks centered at 510 and 690 cm^−1^ can be attributed to Cr–C and Al–N bonding, respectively. More recently, the third region was investigated in ref. [31], where the Raman spectrum of Cr–Al–C was found to contain two peaks at 1316 cm^−^^1^ and 1531 cm^−^^1^, peaks attributed to the D- and G-mode, respectively [31]. These peaks were interpreted as signalling clusters of nanoscale CVD diamonds with wide boundaries of sp2-bonded carbon [31]. This assignation comes once again to confirm our assumption that the formation of D- and G- bands is consistent with carbon nanoclustering within interfacial regions between Cr2AlC hexagonal crystallites. There is also a potential interpretation of the dominance of D- and G-bands attributed to *sp2*-bonded carbon, reported in ref. [32], where it was suggested that large-size carbon clusters have preferential orientation by forming tile-like arrangements [32]. However, it is easy to remark that among the vast amount of both experimental and calculated data reported in the literature and compiled in Table 1, there are no experimental reports, other than ours, that present all three spectral regions, over a large interval, in a unified and integrated manner.

### 3.3. Structural Analysis

**Figure 5:**
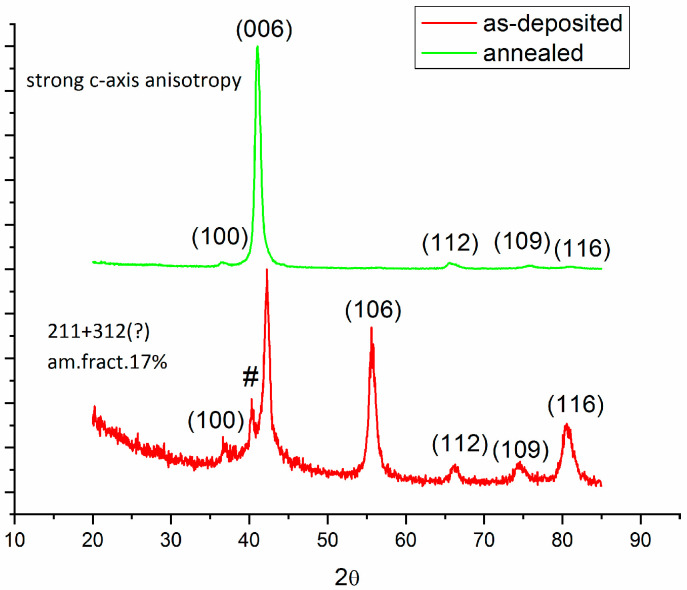
XRD patterns of the films as-deposited (bottom) and films annealed at 700 °C (top).

To identify whether the deposited films of Cr_2_AlC are amorphous or crystalline, it is sometimes customary to record Raman spectra and to check the broadness of the observed Raman peaks. For instance, Ougier et al. [16] presented Raman spectra to confirm the formation of the Cr_2_AlC MAX phase during annealing. It was argued [16] that there are four Raman bands in the first spectral region, between 150 and 400 cm^−1^; however, the second and third contributions overlap and are hardly detectable in the experimental data. A large hump in the Raman spectra observed for the as-deposited film was interpreted by the authors in ref. [16] as proof that the sample is amorphous. After annealing the as-deposited sample, they observed three Raman peaks [16], *1a*, *1b* convoluted with the *1c,* and *1d*, and they attributed this to the partial crystallization of the Cr_2_AlC MAX phase. As one can see from the large literature overview in Table 1, not only is our work the only one presenting all three spectral regions, over a large interval, in a unified and integrated manner, but we have also been able to use our fitting procedure to separate the *1b* and *1c* Raman bands and provide their correctly assigned values in the overview of literature values presented in Table 1. As the broad lines in the Raman spectra can sometimes account for an amorphous-like state of the deposited films, there is also the possibility to confirm and to further prove the structure of the films by means of X-ray diffraction studies. We show hereafter that the results obtained from the fitting refinement of the Raman measurements were further confirmed by structural analysis using X-ray diffraction (XRD). XRD measurements were conducted on the as-deposited and annealed samples in a grazing incidence geometry using a θ–2θ instrumental arrangement, with the beam at an incidence angle of 1.5°. Figure 5 shows the XRD diffractograms recorded for the samples, as-deposited and annealed at 700 °C. Since the diffractogram recorded for the sample annealed at 650 °C is almost identical to the one annealed at 700 °C, we chose to present only one of these diffractograms to have a much clearer image of the net differences between the as-deposited and annealed sample. The XRD pattern of the as-deposited sample shows that the sample is mostly crystalline, as witnessed by the sharp and intense observed Bragg peaks, with the main phase indexed as Cr_2_AlC, while the main Bragg peak of the Cr_3_AlC_2_ phase, a higher symmetry 312 phase also belonging to the MAX phase family, is visible at around 40° in 2θ but with low intensity. Taking into account the results of the full-profile fitting, we found the relative proportion of the amorphous fraction to be approximately 17%. The detection of the amorphous phase was only carried out after subtracting the background of the diffractogram, and the component attributed to the amorphous phase was fitted to fully take into account the broad region situated below the sharp Bragg peaks located at roughly 42°.

In the film annealed at 700 °C, the XRD pattern presented no detectable amorphous fraction in the pattern, the sample being completely nanocrystallized, with the mean grain size increased from 12 nm in the as-deposited case to approximately 42 nm. Interestingly, most of the Bragg peaks of the isotropic structure of Cr_2_AlC, except for the (0 0 3) main peak, strongly diminished or completely disappeared. The strong Bragg reflection due to the (0 0 3) planes provides evidence of the preferential arrangement of the lattice along the c-axis, therefore documenting a strong c-axis anisotropy in the sample annealed at 700 °C. No other peaks, possibly belonging to other binary phases, were observed in the diffractograms of the annealed samples.

In both cases, full-profile analysis of the XRD diffractograms was undertaken. For this purpose, XRD patterns were fitted using Materials Analysis Using Diffraction MAUD software [33] which uses a refinement method similar to the Rietveld full-profile analysis adjusted for topologically disordered alloys by including the function of pair distribution [34]. The deconvolution of Bragg peaks that are broad, such as in the case of amorphous and polycrystalline materials [35], follows the algorithm largely described in our previous papers [36,37,38,39,40,41,42,43,44]. For the as-deposited film, besides the visible sharp Bragg peaks belonging to the hexagonal Cr_2_AlC structure, there was a clear amorphous contribution signalled by the broad bump in the line profile beneath the main Bragg peak, centered at roughly 42°. Full-profile fitting allows correct deconvolution of the broad line, as the crystalline Bragg peaks were all resolved. The relative proportion of the amorphous fraction, determined upon the whole profile MAUD fitting, was approximately 17%. This amorphous fraction is most probably carbon-rich and most definitely responsible for the strong carbon bonding documented in the Raman spectrum, where highly intense Raman peaks associated with the D- and G-bands of carbon were found. In this way, the results from the Raman analysis were fully confirmed by the structural findings from the full-profile fitting of the XRD diffractograms.

For the annealed film, only the sharp Bragg peak assigned to the (0 0 6) reflection of the hexagonal Cr_2_AlC structure occurred in the pattern. The broad bump corresponding to the amorphous fraction was not visible, the baseline of the diffractogram being almost flat. In other words, upon annealing, the sample was completely nanocrystalline, with no quantifiable amorphous fraction being found in the XRD pattern.

All results of the fitting corresponding to the main 211 MAX phase, including the lattice parameters, unit cell volume, the average crystallite size, and the microstrain parameters that were obtained using MAUD, are listed in Table 2.

It is observable that while *a* diminishes with increasing annealing temperature, decreasing by approximately 2%, for the film annealed at 700 °C compared to the as-deposited film, the *c* parameter decreases noticeably (3%) in the annealed case. It is customary in intermetallic alloys that the process of re-crystallization follows the two-step sequence: primary crystallization sometimes followed by secondary crystallization, in modes related to the nucleation and growth model with various activation rates. In the present case, during re-crystallization of the film, pronounced ordering was produced along the *c*-axis, accompanied by the occurrence of Cr_6_C octahedral building blocks stacked alternately to Al layers in order to produce the Cr_2_AlC nano-laminate structure [3]. Unit cell volume also showed a tendency towards diminution after annealing the films. In addition to crystallization, annealing the films at higher temperatures refined the Cr_2_AlC hexagonal lattice, and consequently, this presented fewer crystal defects; therefore, the unit cell volume was smaller than for the as-deposited film.

Following crystallization, the mean crystallite dimension showed an increase from 12 nm, for the as-deposited film, to approximately 38 and 42 nm for the films annealed at 650 °C and 700 °C. It is worth mentioning that, after thermal treatment, the lattice microstrain decreased by roughly 63% compared to the lattice microstrain observed in the as-deposited sample.

### 3.4. Carbon Bonding

The strong carbon bonding documented in the Raman spectrum, arising from the nanoclustering of carbon in the Cr_2_AlC films, requires several clarifications. While investigating the compositional influence on the structural features of CrAlC alloys, previous works [5] established that even though some deviations from the stoichiometry corresponding to the pure 211 MAX phase were detected by experiments, the measured XRD diffractograms did not indicate the presence of phases, other than the 211 phase. According to the CrAlC phase diagram, deviations from 211 stoichiometry may result in the apparition of other binary phases. By combining XRD analysis with ab initio calculations, it was concluded that small deviations from 211 stoichiometry would still result in the occurrence of a single Cr_2_AlC phase. Several potential ways for explaining the missing binary phases on extended compositional ranges are presented in ref. [5]. Among others, this was explained by “segregation of material which escapes detection by XRD, segregation of an amorphous phase or another phase that cannot be detected by XRD”. Previous calculations via ab initio VASP code onto several defect-less Cr_2_AlC structures or Al excess structures were shown to accommodate experimental observations well. In agreement with these modelling results taken from ref. [5], the values we determined for the lattice parameters and the unit cell volume in the film annealed at 700°C, corresponding well with the hexagonal crystal structure and the chemical formula Cr_2_Al_1.1_C. Considering the exact composition that we found in our films, the excess of Al in the structure we obtained after annealing can be explained by the interfacial segregation of carbon, a material hardly observable in XRD. On the other hand, in ref. [31], the two peaks at 1316 cm^−^^1^ and 1531 cm^−^^1^, assigned to D- and G-modes, were interpreted as being due to clusters of nanoscale or diamond-like structures with wide boundaries of sp2-bonded carbon [31]. This hypothesis has indirect evidence also found in ref. [32], where a preferential orientation for carbon clusters is translated into tile-like arrangements at grain boundaries [32]. Therefore, our conclusion, based on corroborating VASP calculations from the literature with our findings, confirms our data and shows without a doubt that this segregated material upon annealing is indeed carbon. Its occurrence is furthermore proven by the observation of the carbon G-band in the Raman spectrum of the film annealed at 700°C. It is thus concluded that UHV annealing of Cr_2_AlC thin films promotes crystal ordering as well as the formation of intergranular carbon nanoclusters/regions within the annealed thin film.

## 4. Conclusions

This work investigated the morpho-structural changes in ternary thin films of composition Cr_47_Al_27_C_26_, which were obtained by ion beam deposition on Si(100) substrates. The films were subsequently heat treated under a high vacuum at temperatures of 650 °C and 700 °C for 30 min. Detailed Raman analysis, powered by a carefully chosen fitting procedure, allowed observation of three different Raman spectral intervals, two of them containing the optical modes that unequivocally identify the low symmetry 211 MAX phase, present in both as-deposited films and annealed films, while the third one indicates the interesting phenomenon of carbon nanoclustering in the intergranular regions between the adjacent Cr_2_AlC 211 phase nanograins. This phenomenon was evidenced by the observation of highly intense Raman peaks attributed to the D- and G-bands of excess carbon in the film. These results were confirmed by the structural data obtained via X-ray diffraction measurements in grazing incidence. The XRD analysis showed that the as-deposited sample was almost completely crystallized in the hexagonal Cr_2_AlC structure. Additionally, a clear amorphous contribution signalled by the broad bump in the line profile beneath the main Bragg peak was identified, centered at approximately 41°. Full-profile fitting using MAUD allowed us to determine the correct deconvolution of the broad line, as the crystalline Bragg peaks were all well resolved. This amorphous fraction of approximately 17% was most probably carbon-rich and responsible for the strong carbon nanoclustering documented in the Raman spectra, where highly intense Raman peaks associated with the D- and G-bands of carbon were found. Both lattice parameters as well as the unit cell volume, were proven to be diminished after annealing as a consequence of grain growth during treatment, a phenomenon which was accompanied by the refining of the structure towards obtaining an almost defect-free lattice.

The average crystallite dimension was proven to increase after annealing, while the lattice micro-strain lowered to approximately 63% in the annealed thin film compared to the as-deposited one. The highly intense Raman lines that were assigned to D- and G-bands of carbon indicate a strong level of order and carbon nano-clustering of Cr_2_AlC thin films.

## Figures and Tables

**Table 1:** Values of the Raman peaks resulted from the fitting of the experimental data, together with their assignations.

Peak No.	1a	1b	1c	1d	2a	2b	2c	3a	3b
As-deposited	176.3	251.3	311.2	347.4	675.2	808.7	-	1364.8	1584.2
Annealed at 650°C	174.5	253.7	307	348.1	684.7	814.4	988.5	1377.4	-
Annealed at 700°C	169.5	254.2	304	347.5	696.6	826.3	992.7	1354.1	1571.9
Literature–experimental [25]	150.9	246.3	n/a	339.2	n/a	n/a	n/a	n/a	n/a
Literature–calculated [26]	160	271	n/a	358	n/a	n/a	n/a	n/a	n/a
Literature–calculated [31]	168	263	n/a	352	n/a	n/a	n/a	n/a	n/a
Literature–experimental [7]	n/a	278	n/a	350	690	n/a	n/a	n/a	n/a
Literature–experimental [31]	n/a	n/a	n/a	n/a	n/a	n/a	n/a	1316	1531
Literature–experimental [16]	165	245	n/a	330	n/a	n/a	n/a	n/a	n/a

**Table 2:** The lattice parameters, the mean crystallite size, unit cell volume and the microstrain of the Cr_2_AlC 211 MAX phase, as revealed by the MAUD fitting of the diffractograms of as-deposited and annealed films.

Sample	Lattice Parameters			Cryst. Size	Microstrain
	a (Å)	c (Å)	V (nm^3^)	(nm)	(%)
As-deposited	2.892 ± 0.014	13.22 ± 0.02	0.096	12 ± 2	0.77 ± 0.04
Annealed 650 °C	2.854 ± 0.007	12.89 ± 0.02	0.091	38 ± 5	0.39 ± 0.03
Annealed 700 °C	2.832 ± 0.005	12.75 ± 0.03	0.088	42 ± 4	0.28 ± 0.03

## Data Availability

The data presented in this study are available on request from the corresponding author. The data are not publicly available due to patenting potential.
